# Acceleration of Batch-type Heterogeneous Ligand-free Suzuki-Miyaura Reactions with Polymer Composite Supported Pd Catalyst

**DOI:** 10.1038/s41598-017-06499-z

**Published:** 2017-08-01

**Authors:** Mian Wang, Han Xue, Fei Ju, Haijie Yang

**Affiliations:** School of Life Science and Technology, Henan Collaborative Innovation Center of Molecular Diagnosis and Laboratory Medicine, Institution Xinxiang Medical University, Jinsui Avenue 601, Xinxiang, 453003 China

## Abstract

An efficient and recyclable palladium (II) catalyst supported on a double-structured amphiphilic polymer composite was reported previously containing a polymer hydrogel within macroporous polystyrene (PS) microspheres. However, some critical questions have been unaddressed. First, the catalyst accelerated the heterogeneous ligand-free batch Suzuki-Miyaura reaction in a H_2_O/EtOH mixture solution at room temperature in the presence of air, which could be ascribed to the “on-water” effect taking place at the interface of the aqueous–organic and basic-aqueous phases created by sodium carbonate in H_2_O/EtOH. To this acceleration, the double-structured amphiphilic polymer composite can also contribute by providing hydrogels inside the macroporous PS that served as a microreactor. This microreactor allowed the reactions to quickly proceed across the two immiscible (i.e. aqueous-organic and basic-aqueous) phases. Moreover, hydrogels containing hydroxyl groups can also serve as phase-transfer catalysts (PTC) to promote the Suzuki reaction. Second, the deactivated catalyst recovered its initial catalytic activity after overnight air exposure. This observation indicates the importance of oxygen in the activation/deactivation of Pd metals, as determined by X-ray photoelectron spectroscopy (XPS) and X-ray diffraction (XRD) measurements which revealed different Pd oxidation states with various morphologies before and after Suzuki reactions.

## Introduction

The development of organic transformations at mild conditions has dramatically improved the environment, economy, and safety of consumers within the last two decades. Transition metal-catalysed C–C cross coupling reactions such as the Suzuki–Miyaura have been pivotal in the development of a variety of important pharmaceutical synthesis routes^1–8^. These reactions typically require high amounts of catalyst (1–10 mol%) and long reaction times (from several hours to a few days)^9–11^, and thereby require safer and greener reaction conditions being both efficient and cost-effective^12–17^. However, only a few methods for carrying out the reaction in an aqueous medium at room temperature have been developed. To circumvent this phase-transfer limitation in the aqueous medium associated with the water-insoluble nature of the substrates, methods based on the utilisation of surfactants, inverse phase-transfer catalysts, supramolecular reactors and water-soluble ligands have been largely used to enhance the reaction rates. However, this area still requires to be further explored.

From an industrial viewpoint, the deposition of Pd nanoparticles on supports is preferred since the obtained catalysts are easier to handle, recover, and re-use within the selected process. Apart from the aforementioned advantages, we believe that these supported Pd catalysts bearing various functional groups can further improve the coupling reaction. For instance, the use of polymers as catalyst supports in continuous flow reactions allows recovering the leached Pd in a similar way as to a chromatography resin. Polymers such as polyethylene oxide (PEO) can be used as phase-transfer catalysts (PTC) to accelerate aqueous-phase Suzuki-Miyaura coupling reactions^18, 19^.

The present study is a follow-up of our previously published communication^20^ containing experimentally based detailed discussions, with some complicated questions left unanswered. In this study, heterogeneous refers to the dissolution of some of the reactants in the reaction mixture to form a suspension. On the other hand, homogeneous refers to the complete dissolution of all reactants in the reaction mixture to form a homogeneous solution. The present study will first examine the accelerated completion of the heterogeneous Suzuki-Miyaura coupling reaction in a H_2_O/EtOH solution in the presence of our polymer composite-supported Pd catalysts (reaction conditions: 0.25 mmol 4-bromobenzaldehyde, 0.275 mmol phenyl boronic acid, 1 mL H_2_O/EtOH (v/v 1:1), 0.01–1 mmol% Pd, room temperature in the presence of air and 5–10 min for reaction completion). In contrast, the homogenous solutions with all reactants dissolved (under the same reaction conditions, except for 10 mL H_2_O/EtOH (v/v 1:1), 0.06 mol% Pd) required more than 6–8 h to complete the coupling reaction^20^. Additionally, this study will try to answer the following questions: (i) Why is not possible to reuse the catalyst right after a Suzuki coupling reaction? and (ii) Could the catalyst recover its initial catalytic activity after being kept under air overnight or longer? To provide answer to these questions, we conducted Suzuki coupling reactions with bromoarenes bearing electron withdrawing and donating groups under batch and continuous-flow reaction conditions. Several reaction parameters (e.g. reactant concentrations, water to ethanol ratio and Pd loading) were investigated to tentatively disclose the underlying mechanism of the accelerated Suzuki reactions while also elucidating the role of the polymer support in this process.

## Results and Discussion

### Kinetics of aqueous heterogeneous batch reactions

**Figure 1:**
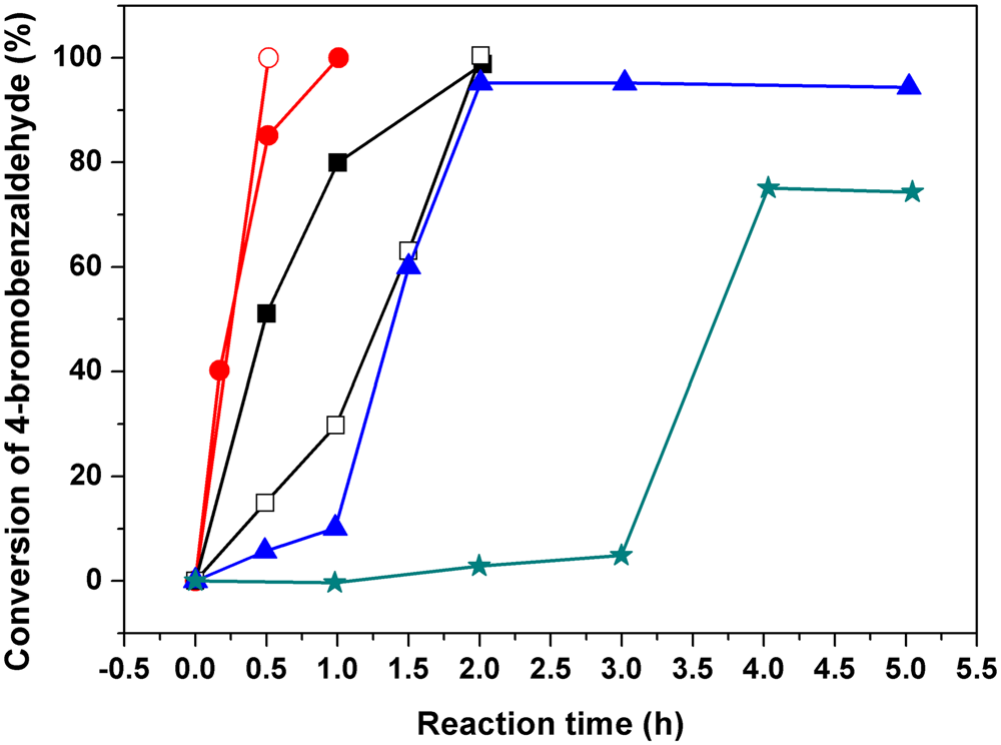
Kinetics of the heterogeneous coupling of 4-bromobenzaldehyde with phenyl boronic acid catalysed by Pd catalyst PCS1 at Pd loadings of 0.006 mol% (red empty circle) and 0.001% mol (black empty square), by PCS2 at Pd loadings of 0.1 mol% (red solid circle) and 0.01 mol% (black solid square), by Pd/C (1 mol%, blue solid triangle), and by PdEnCat^TM^40 (1 mol%, green solid star) under concentrated batch condition. (4-bromobenzaldehyde 0.25 M, phenyl boronic acid 0.275 M, H_2_O/EtOH (v/v 1:1, total volume 1 mL) at room temperature without argon protection.

Heterogeneous aqueous reactions are advantageous in that they allow high reactant concentrations and simple product isolation while also being safer and less costly as compared to those carry out in organic solvents. As previously reported, our polymer composite-supported Pd catalyst could be used for ligand-free Suzuki-Miyaura coupling reactions at room temperature. First, the catalytic efficiencies of PCS1 and PCS2 were compared with those of two commercial polymer composite-supported Pd catalysts (Pd/C and PdEnCat^TM^40) under heterogeneous reaction conditions. The Pd loading used was 1 mol% for the two commercial catalysts. At this Pd loading, PCS1 and PCS2 showed extremely fast coupling reaction rates and therefore their results were not included herein. In this sense, the Suzuki coupling reaction of 4-bromobenzaldehyde with phenyl boronic acid is typically completed in less than 5 min with PCS1 at a Pd loading greater than 0.01% (1 mol% for PCS2). As shown in Fig. 1, PCS1 is the most efficient supported Pd catalyst able to complete the reaction in 30 min and 2 h at only 0.006 and 0.001 mol% Pd, respectively. In contrast, commercial Pd/C at 1 mol% Pd showed a conversion of ca. 95% after 2 h, while PdEnCat^TM^40 with the same Pd loading reached 78% conversion after 4–5 h of reaction. Additionally, an induction period was observed for the two commercial catalysts (ca. 1 and 3 h for Pd/C and PdEnCat^TM^40, respectively), thereby revealing some adverse effects of the reactant mixture over the supported Pd active sites. In contrast, no induction period was observed for PCS1 and PCS2. Instead, the conversion of the reactant proportionally increased with the reaction time during the first half of the catalytic process at various Pd loadings. Additionally, we compared the research data disclosed recently on various Pd catalysts for aqueous Suzuki reactions (see Supplementary Table S1), and concluded that our polymer composite supported catalyst predominantly showed superior catalytic efficiencies while being more environmentally friendly.

### Kinetics of Aqueous Homogenous Batch Reactions

**Figure 2:**
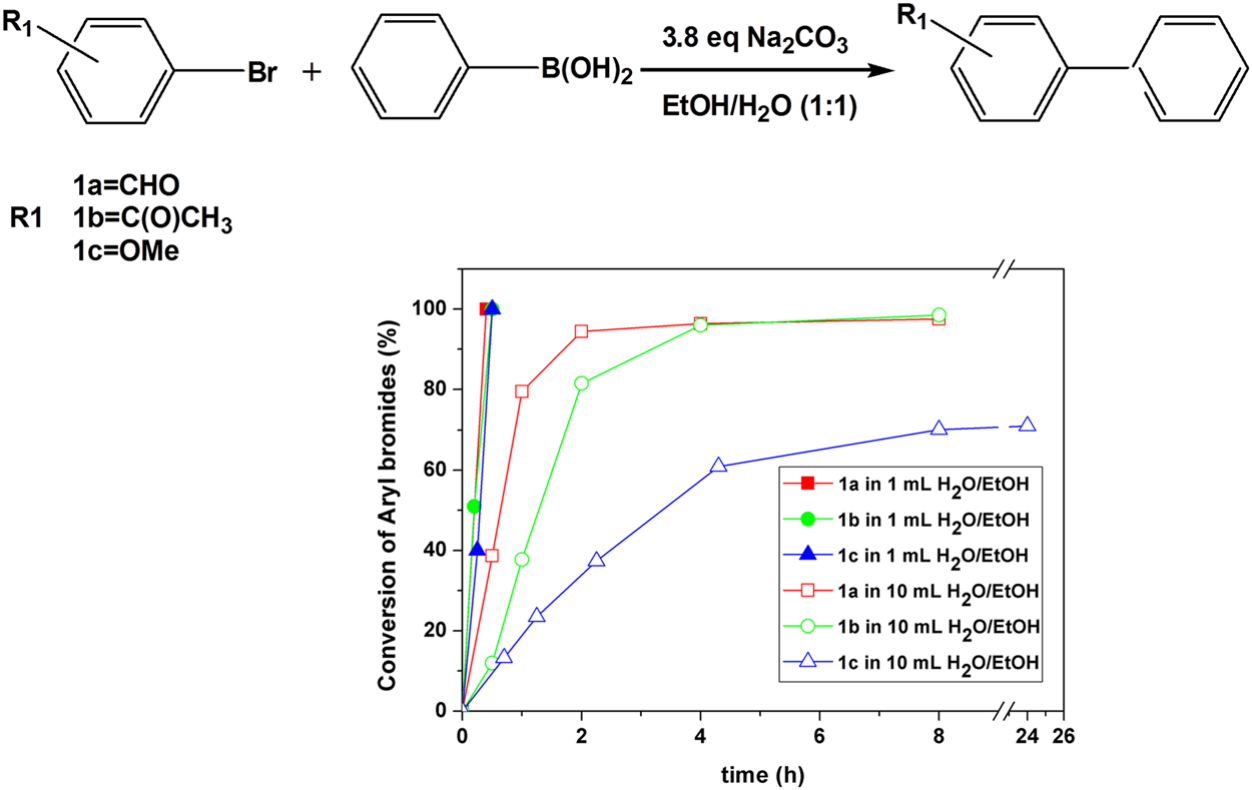
Conversion of aryl bromides (0.25 mmol) at different intervals in the batch Suzuki-Miyaura coupling of phenyl boronic acid (0.275 mmol) in H_2_O/EtOH (1:1 v/v) under heterogeneous (1 mL) and homogeneous conditions (10 mL) at room temperature. Concentrations of aryl bromides and phenyl boronic acid are 0.25 M and 0.275 M, 0.025 M and 0.0275 M, respectively. The Pd loadings with respect to aryl bromides are 0.006 mol% and 0.06 mol%, for concentrated and dilute reactions, respectively.

With the aim to confirm whether this ultrafast nature of the Suzuki reaction could be achieved under homogenous/dilute batch conditions, we investigated the conversion of bromoarenes with both electron-withdrawing and electron-donating functional groups as a function of time. As shown in Fig. 2, PCS1 (Pd loading of only 0.006 mol%) under heterogeneous conditions showed coupling rates in the following order: 1a > 1b > 1c. Under homogenous conditions (i.e. 10-fold diluted reactant concentration and Pd loading increased 10-fold to 0.06 mol%) the Pd catalyst 1a and 1b showed conversion higher than 98% within 8 h in the presence of electron-withdrawing groups, and this conversion decreased to ca. 70% in the case of 1c (bearing an electron-donating functional group) and remained constant with time. These results summarizing the effect of various functional groups are in agreement with those previously published in the literature^21^. However, the dilution employed might also contribute to decrease the reaction rate. This might indicate that the reaction did not take place in the aqueous phase since the reaction rates “in-water” are typically faster under dilute conditions^22^. Thus, we propose that the ultra-fast Suzuki coupling observed herein might occur at the interface of the solvent mixture (i.e. “on-water” reactions)^23^.

### On-water effect on the acceleration of the Suzuki–Miyaura coupling reaction under heterogeneous batch conditionsn

**Figure 3:**
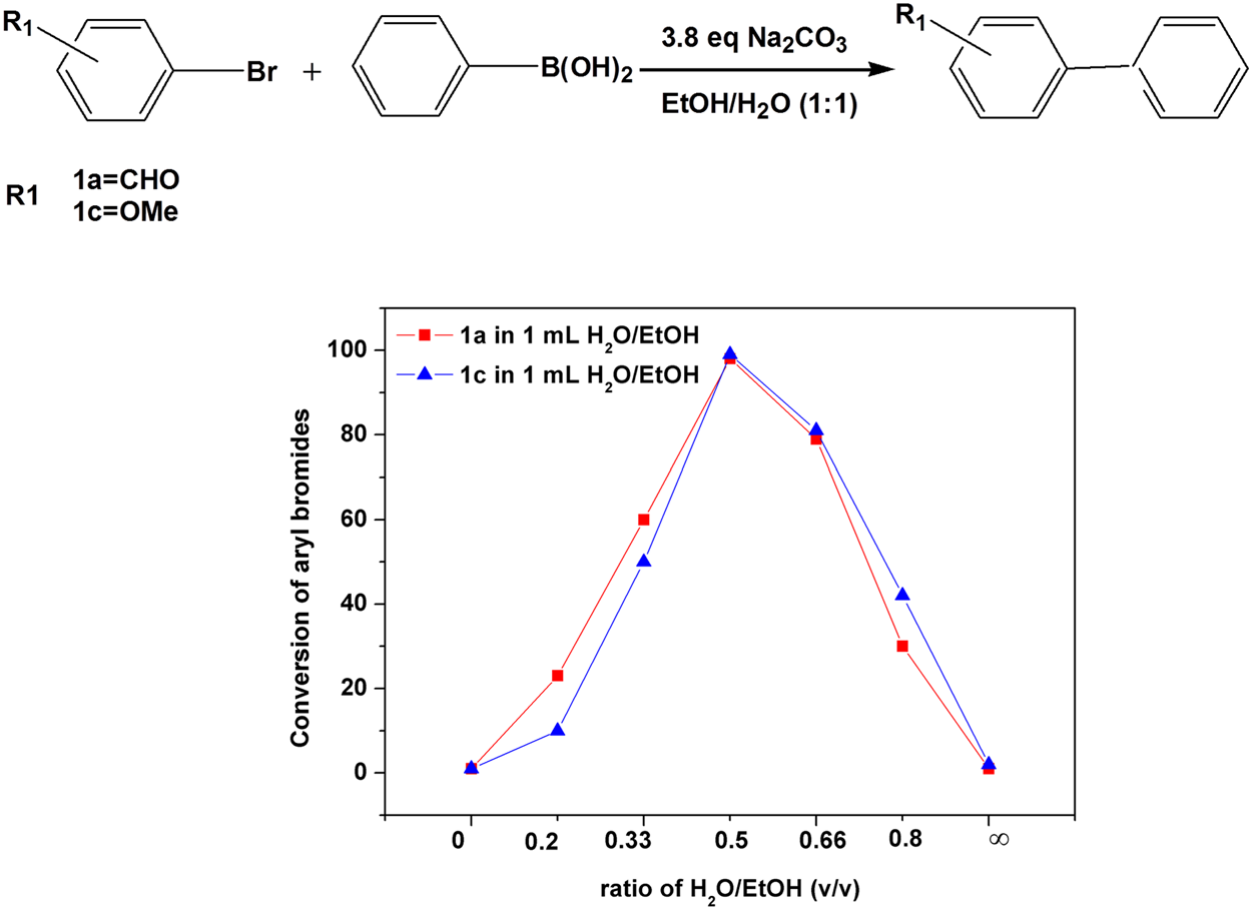
Investigation of the influence of the ratio H_2_O/EtOH on the conversion of aryl bromides (0.25 M) in batch Suzuki-Miyaura coupling, with phenyl boronic acid (0.275 M) in H_2_O/EtOH under concentrated condition in 10 min at room temperature in air with PCS1 at 0.01 mol% Pd loading with respect to the aryl bromides.

The causes behind the rate acceleration in homogenous water-based reactions have been extensively investigated^24^. In the case of heterogeneous aqueous reactions, several factors such as the concentration of the reactants, the water/organic solvents ratio, the phase splitting of the reaction media, and the stirring speed might account for the observed on-water acceleration effect. In heterogeneous Suzuki coupling reactions, a microscopic phase separation is present between two phases: aqueous-organic phase with lower pH and aqueous basic phase with higher pH^25^. Phenyl boronic acid is ionized in the aqueous basic phase. Thus, unlike the positive ions that are repelled from the interfaces, phenyl boronic acid is present mostly in excess at the interfaces of the two phases^26^. On the other hand, organic acryl bromide molecules should be present in the aqueous–organic phase such that the coupling mainly occurs at the organic–water interface rather than in the organic phase. As shown in Fig. 3, 0.25 M solutions of 1a (electron-withdrawing group) and 1c (electron-donating group) were hardly converted when in a total volume of reaction of 1 mL of pure ethanol or water. However, the coupling rates increased with the ethanol to water (v/v) or water to ethanol ratios up to 0.5.Notably, two parameters might influence the reaction rates provided the Suzuki coupling reactions take place at the water–ethanol interface: a) the solubility of the reactants in ethanol (aryl bromides) and water (phenyl boronic acids and sodium carbonate); and b) the interface area between the aqueous–organic (acryl bromide/ethanol) and the basic aqueous (containing phenyl boronic acid and sodium carbonate) phases. The former is determined by the relative amounts and ratio of the solvent mixture, whereas the latter is influenced by solvent mixture ratio and the stirring intensity. In our case, although ethanol is miscible in water, the addition of sodium carbonate induced phase splitting, as commonly observed for water-miscible solvents such as tetrahydrofuran (THF), dioxane and methanol typically employed for the Suzuki-Miyaura coupling reaction. The extended UNIQUAC model^27^ previously described in detail this liquid–liquid split of the aqueous ethanol solution containing Na_2_CO_3_. As for the on-water reactions, the difference between the homogenous and heterogeneous reactions can be attributed to the reaction occurring at the extensive aryl bromide/ethanol oil droplet interfaces, these reactions being significantly faster than those in the bulk. For a given total amount of reactants, the rate of the surface reaction is inversely proportional to the size of the droplets since small droplets have larger total surface area-to-volume ratios^28^. Obviously, in dilute homogenous reactions, the surface area of the aryl bromide/ethanol-water mixture is very low or nearly zero, thereby allowing the Suzuki coupling reaction to proceed in the homogenous bulk solution without the occurrence of the on-water acceleration process and explaining the low conversion of bromoarenes in pure ethanol and water (Fig. 3). The interface area may reach a maximum at water/ethanol ratios close to 1, thereby allowing the on-water effect. This phenomenon was also described by Sharpless *et al*. for other organic transformations. These authors observed significantly lower reaction times (by a factor of 300) for a heterogeneous mixture of reactants on water as compared to a homogenous solution of reactants in water^29^. From the viewpoint of the molecular reactions, the -OH groups of water play a vital role in the on-water catalysis. At the emulsion/heterogeneous interface, some OH groups can migrate into the organic phase being readily available to catalyse the reactions. In the homogenous solution, the existing H-bond network from the water molecules surrounds the reactant before releasing the OH groups to catalyse the reactions^28, 30^. This process requires both time and energy, thereby decreasing the rate of the homogeneous reactions versus the heterogeneous reactions.

### Mechanism and role of the polymer support in the on-water Suzuki–Miyaura reactions

To characterize the role of the support in this unique on-water acceleration reaction, we compared its catalytic activity with those of commercial supported catalysts (i.e. Pd/C and PdEnCat^TM^40), and the results are summarized. (see Supplementary Table S2). Pd/C and PdEnCat^TM^40 also catalysed the Suzuki coupling reaction at room temperature, and moderate yields of the products were obtained within one to several hours. According to the merchant’s website, PdEnCat^TM^40 can convert 94% of 4-bromoanisole to the desired product within 8–12 h in a toluene/water/ethanol (4/2/1) solvent mixture at an elevated temperature of 80 °C ^31^, which indicates the importance of a proper solvent selection. In terms of the role of the different supports, activated carbon in Pd/C might only serve as a support since the inner walls of the reaction flask were observed to have a black film of Pd metal after the Suzuki coupling reaction. In contrast, no precipitation of black metal was observed during the entire coupling process for both PdEnCat^TM^40 and polymer composite-supported PCS1 and PCS2 catalysts. PdEnCat^TM^40 was supported on a macroporous polyurea matrix, whereas the supports of PCS1 and PCS2 consisted of soft hydrophilic polymer gels with hydroxyl or amide functionalities. Therefore, these polymeric matrices functioned as a support while also interacting with Pd and preventing it from agglomeration or deactivation processes. However, PdEnCat^TM^40 showed an induction time and did not accelerate the heterogeneous Suzuki reaction (Fig. 1), thereby implying that hydrophobic polyurea is less effective than the hydrophilic hydrogels of PCS1 and PCS2. We also propose that the use of hydrophilic PHPA gels as PTCs can contribute to the on-water acceleration of the Suzuki coupling reaction.

Polymers have been widely used as PTC. For instance, PEG has been often utilised as a PTC in a variety of processes (e.g. Suzuki coupling reactions^18^), given the ability of the poly(ethylene oxide) (PEO) chains to form complexes with the metal ions^19^ while also being capable of transferring active ArB(OH)^3−^ species from the aqueous to the organic phase^18^. The mechanism is that the oxygen in the PEO chains accepts H^+^ from phenylboronic acid to maintain the electron neutrality of the surrounding environment. Likewise, ArB(OH)^3−^ species can also passively accumulate around the polymer chains.

**Figure 4:**
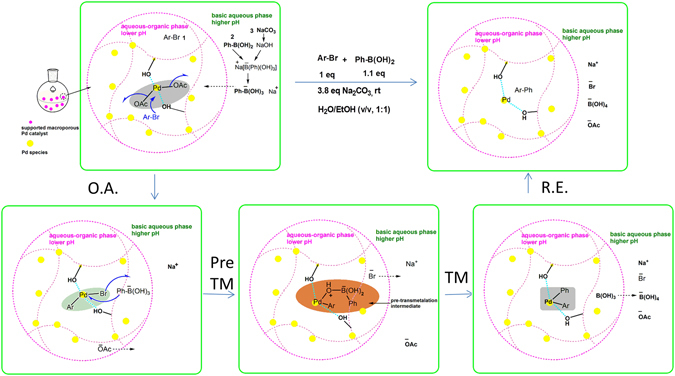
Mechanism of heterogeneous Suzuki-Miyaura coupling catalysed by polymer composite supported Pd catalyst PCS1.

Likewise, in the case of PCS1, numerous -OH groups from the PHPA hydrogel also attract H^+^, thereby allowing the migration of ArB(OH)^3−^ species from the basic aqueous to the aqueous–organic phase and accelerating the Suzuki coupling reactions. The chemical nature of the supports in PCS2 and PdEnCat^TM^40 (i.e. amide groups) did not increase the coupling reaction rate to the same extent of PCS1. Furthermore, the amphiphilic nature of the polymer composite supports as well as their three-dimensional network resulted in swollen hydrogels that facilitated the diffusion of different reactants and product molecules in and out of the macropores. These polymer chains with a hydrated conformation (in case of hydrophilic polymers) were previously observed to present maximum catalytic activity^32^. A possible mechanism for the heterogeneous batch Suzuki coupling reaction over PCS1 is depicted in Fig. 4 that considers the above discussions and theories.

### Kinetics of aqueous homogenous continuous flow reactions

**Figure 5:**
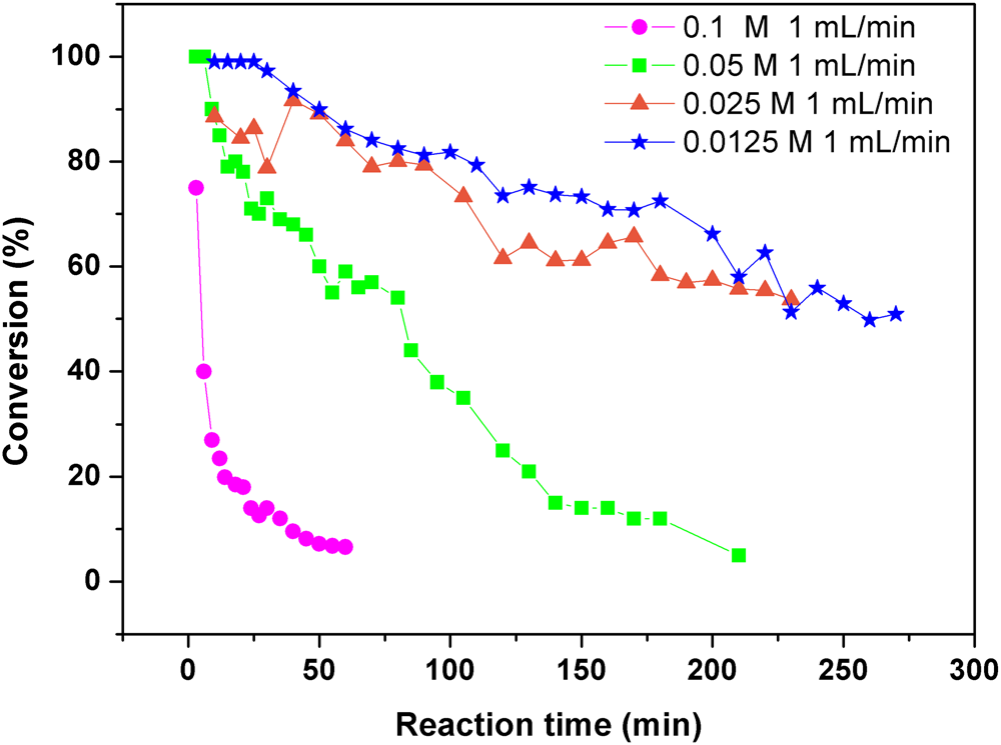
Conversion of 4-bromobenzaldehyde in Suzuki-Miyaura coupling with phenyl boronic acid in H_2_O/EtOH at room temperature in air. Concentrations of 4-bromobenzaldehyde was in a range of 0.0125–0.1 M; Pd catalyst loading ca. 0.033 mmol; flow rate 1 mL/min.

Supported Pd catalysts can be used for Suzuki coupling reactions at room temperature without the addition of ligands and inert gas protection. Thus, these catalysts are more suitable for large scale continuous flow reactions at the industrial scale. However, the longevity and stability of these catalysts require further testing. The apparatus employed for the flow reaction is depicted (see Supplementary Fig. S3). The PCS1-catalysed coupling reaction between 4-bromobenzaldehyde and several phenyl boronic acids was monitored with the conversion of 4-bromobenzaldehyde as a function of time (Fig. 5). Nearly 99% of the reactant was converted within 3–40 min, and this conversion was higher than 80% within 100 min (residence time: ca. 3 min; Pd loading: 0.033 mmol, concentration: 0.0125 M), thereby suggesting the high efficiency of the supported catalyst. Conversion moderately decreased with time (>50% for both 0.0125 and 0.025 M 4-bromobenzaldehyde solutions within 230 min). As expected, further increasing of the concentration to 0.05 and 0.1 M led to more severe and progressive deactivation of the supported Pd catalyst. Thus, higher concentrations of reactants have opposite effects on the heterogeneous batch and homogenous continuous flow reactions, and obviously no “on water” effect was observed in the continuous flow reactions. This can also be ascribed to the homogeneity of the reaction mixture as explained for homogeneous batch reaction.

### Identification of the active Pd species in Suzuki coupling reactions

The true nature of the active Pd species in heterogeneous Suzuki coupling reactions was also investigated by X-ray photoelectron spectroscopy (XPS) measurements. The asymmetric shapes of the Pd 3d bands in all the XPS spectra (see Supplementary Fig. S4) suggest the presence of several Pd species in the catalytic system. Two pairs of peaks with different intensities were observed after the deconvolution of the curves. The binding energies (BE) of the Pd 3d bands of PCS1 before and after the Suzuki and Heck reactions under different reaction conditions are summarized in Table 1. In the case of the as-prepared PCS1, the presence of double peaks at ca. 337.2 (Pd_3d 5/2_) and 342.5 eV (Pd_3d 3/2_) confirmed the successful immobilization of Pd(II) on the polymer composite support^33^. After the Suzuki and Heck reactions, large amounts of Pd(II) (>60%) were *in situ* reduced to Pd(0), and this was accompanied with a colour change in the catalyst (from light yellow to grey). Although only two Pd species were studied herein, the presence of “electron deficient” Pd species (Pd^δ+^, 0 < δ < 1) cannot be ruled out^34^. The strong interaction between the metal and oxygen atoms on the supports resulted in XPS spectra revealing higher metal oxidation states^35^. As stated in our previously published report, PCS1 and PCS2 were unsuccessfully reused after successive Suzuki coupling reactions. However, we further discovered that keeping the used catalysts under air overnight or longer was beneficial to recover the initial activity of the catalysts for Suzuki coupling reaction cycles. An XPS analysis of the Pd species revealed that a large fraction of Pd(0) was oxidized to Pd(II)/Pd^δ+^ under air as previously reported by two independent research findings^36, 37^. For example, the Pd(0) species sequestered within G4-OH were gradually oxidized to Pd(II) with time under laboratory atmosphere^37^. Remarkably, no isolated PdO species were observed, thus it was hypothesized that the Pd ions were coordinated to/with the dendrimer internal structure. Furthermore, this oxidative reaction was reversible, as the oxidized Pd nanoparticles were found to be reduced to Pd(0) by either hydrogen gas or during the Suzuki coupling reaction^38^. In our study, the high amounts of Pd(0) produced after the first Suzuki reaction were catalytically inactive. In contrast, used PCS1 containing a high fraction of Pd(II)/Pd^δ+^ species upon air oxidation was catalytically active. Thus, Pd(II)/Pd^δ+^ (Pd in higher oxidation states) are proposed to favour the Suzuki coupling reactions, whereas both Pd(0) and Pd(II) are the catalytically active species for the Heck reaction. Even the Pd(0) species were formed after both the reactions, they may present different physical properties and therefore different catalytic activities, which will be discussed in the following part.

**Table 1:** Oxidation states and ratios of different Pd species.

Catalyst PCS1	BE of Pd(0) eV	BE of Pd(II) eV	Ratio of Pd(0)	Ratio of Pd(II)/Pd^δ+^
as-prepared PCS1	334.8 340.3	337.2 342.5	26.5%	73.5%
after 1^st^ heterogeneous Suzuki coupling	335.0 340.3	337.3 342.6	76.6%	23.4%
kept in air after 1^st^ heterogeneous Suzuki	335.0 340.3	336.8 342.1	41.1%	58.9%
after 1^st^ homogeneous Suzuki coupling	334.6 339.9	337.0 342.3	67.8%	32.2%
after continuous flow Suzuki coupling	334.5 339.8	336.8 342.1	64.7%	35.3%
after 1^st^ Heck reaction	335.6 340.9	337.5 342.8	85.4%	14.6%

SOS for Pd_3d 3/2_ and Pd_3d 5/2_ is set to 5.3 Ev, FWHM is 2.3 and 2.5 for Pd(0) and Pd(II), respectively. And the ratio of peak area of Pd_3d 5/2_ to Pd_3d 3/2_ is 1.5.

### Crystalline structure of the supported Pd catalyst

**Figure 6:**
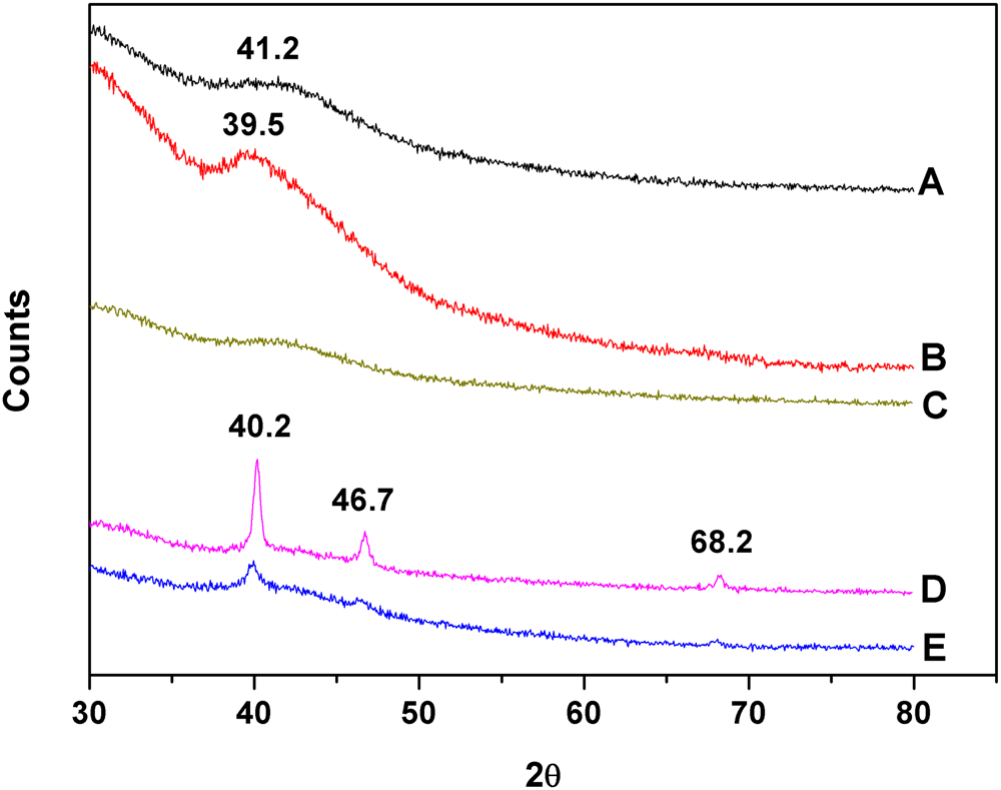
XRD patterns of polymer composite supported Pd catalyst PCS1: (A) as-prepared polymer composite supported Pd (II) catalyst. (B) After 1^st^ heterogeneous batch Suzuki reaction of 4-bromobenzaldehyde with phenyl boronic acid; (C) kept in air after 1^st^ heterogeneous batch Suzuki reaction; (D) after 1^st^ Heck reaction; (E) after 10^th^ Heck reaction of iodobenzene with butyl acrylate.

PCS1 was analysed before and after the Suzuki and Heck reactions by X-ray diffraction (XRD). It is well known that the crystalline structure of the metal is important to its catalytic activity. For instance, the catalytic selectivity of Pt in the benzene hydrogenation reaction was found to be strongly affected by the shape of the nanoparticles^39^. As shown in Fig. 6A, broad diffraction peaks centred at ca. 2θ = 41.2° can be ascribed to amorphous Pd(II) on as prepared PCS1, which is in agreement with the XPS results. After 1st Suzuki coupling reaction, PCS1 showed only one broad peak at ca. 39.5°, which is a characteristic of amorphous metallic palladium Pd(0) (Fig. 6B). After air oxidation, used PCS1 showed a broad peak (Fig. 6C) similar to that for Pd(II) (Fig. 6A) with reduce intensity. In contrast, the diffractogram of the catalyst after 1st Heck reaction showed one sharp peak at *ca*. 40.2° and two weak peaks at 46.7 and 68.2°, which correspond to the (111), (200), and (220) crystallographic planes of the face-centred cubic crystalline (fcc) phase of Pd metal (Pd(0))^40^. The (111) peak was significantly more intense than that of the (200) plane, indicating randomly oriented three-dimensional assembly of the Pd crystals^41^. The intensities of the three peaks of crystalline Pd(0) decreased after the 10th Heck reaction, thereby suggesting partial loss of the ordered structure during the cycle reaction. Thus, the different morphology of the Pd(0) species in PCS1 after the 1st Suzuki and 1st Heck reaction also contributed to its different recyclability in the both reactions.

### Change in the surface of the polymer composite

In the design of our polymer composite support, the macroporous hard polymer skeleton PS provided a structural protection for the soft gels PHPA and PDMAA. These gels grow and interpenetrate within the pores of the skeleton, thus leading to a three-dimensional network. The different swellabilities of the gels in aqueous and organic solvents and the morphological change of the support during the organic transformations may have also influenced their interactions with the supported metal catalysts and consequently their catalytic activities. Thus, the surface composition of the support was examined by XPS. The carbon to oxygen (C/O) atomic ratios were calculated as follows: N_C_/N_O_ = (I_C_/S_C_)/(I_O_/S_O_)^42^. In the case of PCS1, the C/O atomic ratio for the support remained nearly unchanged after the first Suzuki coupling reaction (1.98 versus ca. 2.03 for pure PHA), thereby implying that the surface of the support surface was mainly covered with PHA hydrogels in the aqueous solution. This value increased to ca. 2.3 after the first and tenth Heck reactions in n-methyl-2-pyrrolidone (NMP), which suggests the hydrophilic moieties in the organic solvent such as hydroxyl groups removed from the support surface by retracting inside the deeper PS macropores. To confirm this, pore properties of PCS1 before and after the both coupling reactions were also measured with BET and results summarized (see Supplementary Table S5). Obviously, after the Heck reaction, the pore volume and diameter were reduced substantially in NMP, whereas the pore property of the support was slightly changed in H_2_O/ethanol for the Suzuki reaction. On the other hand, the peak intensity ratio of Pd 3d to C1s was 0.195 after the first Suzuki coupling reaction (versus 0.084 for the as-prepared PCS1 and 0.023 after the first Heck reaction). These results imply the presence of a more significant number of Pd ions on the support surface or, alternatively, the formation of larger Pd nanoparticles during the Suzuki reaction as compared with the Heck reaction process.

## Conclusions

We herein addressed the questions related to the efficiency and recyclability of Pd(II) catalyst supported on a double-structured amphiphilic polymer composite. The observed rate increase of the heterogeneous ligand-free Suzuki-Miyaura reaction in water/ethanol at room temperature in the presence of air has been scarcely investigated in the literature. We ascribed this difference between the heterogeneous and homogenous reactions to the on-water effect taking place on the interfaces of the aqueous-organic (at low pH) and the basic aqueous (at high pH) phases which was discussed in detail. The hydrogels contained in the polymer composite, along with its hydroxyl groups, can also serve as PCT in organic transformations promoting the Suzuki reaction. Additionally, the deactivated catalyst in the Suzuki reactions can recover its initial catalytic activities after an overnight air treatment. XPS and XRD studies revealed the presence of different Pd oxidative states and various Pd morphologies before and after the Suzuki and Heck reactions, with oxygen likely playing a key role in the activation and deactivation of the Pd metal. In summary, this double-structured polymer composite-supported Pd material is an efficient and cost-effective catalyst for performing green Suzuki reactions.

## Experimental

### Materials

Pd(OAc)_2_ (99%, Sigma-Aldrich), phenylboronic acid (97%, Fluka), 4-bromobenzaldehyde (99%, Alfa Aesar), 4-bromoanisole (99%, Alfa Aesar), were used as received. The synthesis of polymer composite supported Pd catalyst was described in literature^11^. Pd(OAc)_2_ catalyst was supported on polymer composites containing polystyrene (PS) macroporous spheres and polymer hydrogels, with poly(2-hydroxypropyl acrylate) being denoted as PCS1 (Pd loading 0.065 mmol/g), and poly(N,N-dimethylacrylamide) as PCS2 (Pd loading 0.075 mmol/g). Pd/C (palladium on activated charcoal, 10% Pd basis, 0.939 mmol/g) and PdEncat^TM^40 (0.4 mmol/g) were purchased from Sigma-Aldrich and used as received.

^**1**^ **HNMR** measurement was conducted on an Avance III spectrometer (400 MHz, Bruker, Germany). Briefly, the final experimental mixture was extracted with dichloromethane (DCM) and the organic layer was collected and vacuum dried. Then the product was dissolved in deuterated chloroform (CDCl_3_) for ^1^HNMR measurements using TMS as internal standard, and conversions were determined on the bases of aryl halides.

**X-ray Powder Diffraction (XRD)** analysis was conducted on a Bruker D8 Advance X-ray diffractometer with Cu K_α_ radiation. The samples were mounted in a low background sample holder and scanned at a rate of 0.02 step^−1^ over the 80° ≥ 2 ≥ 30° range with a scan time of 5 s step^−1^. The diffractograms were compared with the references for identification purpose.

**X-ray Photoelectron Spectroscopy (XPS)** measurements were performed on an ESCALAB 250 spectrometer, using Al Kα radiation (1486.6 eV, pass energy 20.0 eV). The base pressure of the instrument is <2.0 × 10^−8^ mbar. A Shirley background was applied to subtract the inelastic background of core-level peaks. The model peak to describe XPS core-level lines for curve fitting was a product of Gaussian and Lorentzian functions. Charge effects were corrected by using the C 1s peak at 285.0 eV. The instrument was also calibrated by using Au wire. XPS spectra were recorded at 90°.

**Brunauer-Emmett-Teller (BET)** test was performed on a surface area & pore size analyser (Beckman SA3100, USA) with N_2_ adsorption at 77 K.

**Inductively Coupled Plasma Atomic Emission Spectrometry (ICP-AES)** test: The Pd content of the catalysts was measured on an IRIS intrepid spectrometer (Thermo Scientific, USA). For typical sample preparation, ca. 20 mg of supported Pd catalyst was reflux in 2 mL concentrated acids (HCl:HNO_3_ = 3:1 v/v). After the solid was completely decomposed, the clear solution was diluted to 10 mL in volumetric flask with DI water. Characteristic wavelengths of 229.651, 340.458 and 360.955 nm were monitored and linear calibration curves were generated.

### Batch and Continuous-flow Suzuki Cross-coupling Reactions

For batch-type reactions, a two-neck round flask was loaded with phenyl boronic acid (0.275 mmol), acryl bromides (0.25 mmol), Na_2_CO_3_ (0.95 mmol), Pd catalyst (0.001–1 mol%) and 1 mL of H_2_O/EtOH (v/v 1:1). The reaction mixture was vigorously stirred for a given reaction time. The reaction mixture slurry was subsequently diluted with 5 mL of H_2_O and 5 mL of dichloromethane (DCM) and filtered. The organic phase was separated and the aqueous layer was extracted twice with 5 mL of DCM. The combined organic layers were washed with 5 mL of water, and the organic solvent was removed under reduced pressure.

For the continuous flow reactions, a homogenous solution containing 4-bromobenzaldehyde, phenyl boronic acid and sodium carbonate in a H_2_O/EtOH (1:1) mixture at given concentrations was pumped through a 20 mL syringe using a PHD 2000 syringe pump (Harvard Apparatus, UK) set at predetermined rates. During the reaction, approximately 30 drops of the product sample were collected at certain time intervals and subsequently extracted with 1 mL DCM. The conversion and yields were determined in the same way as mentioned previously.

### Data availability statement

The authors declare all data available in manuscript and supporting information files.

## Electronic supplementary material

Supplementary Information
