# Supplementary material for: Acceleration of Batch-type Heterogeneous Ligand-free Suzuki-Miyaura Reactions with Polymer Composite Supported Pd Catalyst
Source: Sci Rep. 2017 Aug 1;7:7006. doi: 10.1038/s41598-017-06499-z (PMC5539156; doi:10.1038/s41598-017-06499-z)
Supplement: Supplementary file 1 — Supplementary Information [file 41598_2017_6499_MOESM1_ESM.pdf]

Supporting Information

Acceleration of Batch-type Heterogeneous Ligand-free Suzuki-Miyaura Reactions with Polymer Composite Supported Pd Catalyst

Mian Wang[1], Han Xue[1], Fei Ju[1], Haijie Yang[1*]

[1]School of Life Science and Technology, Henan Collaborative Innovation Center of Molecular Diagnosis and Laboratory Medicine, Xinxiang Medical University Jinsui Avenue 601, Xinxiang, China 453003

*Correspondence to: Haijie Yang

E-mail: 131034@xxmu.edu.cn

Supplementary Table S1 Comparison of the activity of different supported catalysts in aqueous Suzuki cross-coupling of aryl bromides.

Catalyst	Pd (mol%)	Reaction conditions	Yield (%)	Ref.
PCS1	0.01	H_2O/EtOH, Na_2CO_3, rt, 5 min, in air	>99	this study
SWCNT-DETA/Pd(II)	1	H_2O/EtOH, K_2CO_3, 60ºC, 2h	90	1
PMPC40-b-PDPAEMA70	10	H_2O/EtOH, K_2CO_3,50 ºC, 4h	80	2
Pd^{2+}/MB-2000-LTA	1.5	H_2O/PEO, K_2CO_3, microwave 150 ºC, 10min	97	3
PS-bis-thiopseudourea-Pd(II)	0.5	H_2O/MeOH, LiOH, rt, 8h	94	4
Melamine-Pd(II)	0.1	H_2O, Na_2CO_3, 80 ºC, 30 min	92	5
$Pd(OAc)_2$	1	H_2O/EtOH, K_2CO_3, r.t., 2h, in air, potassium aryltrifluoroborate	95	6
Styrene-DVB supported Pd(0)	10	H_2O/EtOH, KOH, rt, 3h,	56	7
Fe3O4/P(GMA-AA-MMA)-Pd(II)	0.2	H_2O/EtOH, K_2CO3, 80 ºC, 2h, in air	99	8
Pd(II) @bbp-MOP	0.27	H_2O/EtOH, K_2CO_3, 80 ºC, <0.25h	99	9
Fe_3O_4/PPy-Pd(0)	1.0	H_2O, K_2CO_3, 70 ºC, 40 min	86.4	10
PVP-capped PdNPs	1.6	H_2O/THF, K_2CO_3, 80 ºC, 48h, 4-iodoanisole	98	11

Pd NPs	10	H_2O, Na_2CO_3, 80 ºC, 24h, phenyl iodide, TBAB	97.1	12
PVP stabilized Pd NPs	0.0006	H_2O/THF, K_3PO_4, 90 ºC, 12h, N_2	91	13
Fe_3O_4/SiO_2-Met-$Pd(OAc)_2$	0.14	H_2O/EtOH, K_2CO_3, 80 ºC, 30 min	97	14
PVI-PVCL/$PdCl_2$	1	H_2O/EtOH, K_2CO_3, 80 ºC, 45 min	97	15
$Na_2Pd_2Cl_6$ complex	0.2	H_2O/MeOH, K_2CO_3, rt, 10 min-2h	37-100	16
Crystalline Pd NPs	0.1-5	H_2O/EtOH,K_2CO_3, rt, in air, aryl iodides, 9.5h	100	17
$Pd(OAc)_2$	1	H_2O/PEG, Na_2CO_3, 50 ºC, 30 min	97	18

References

[1] Ghorbani-Vaghei， R., Hemmati, S., Hashemi, M. & Veisi, H. Diethylenetriamine-functionalized single-walled carbon nanotubes (SWCNTs) to immobilization palladium as a novel recyclable heterogeneous nanocatalyst for the Suzuki–Miyaura coupling reaction in aqueous media. *C. R. Chim.* **18**, 636-643 (2015).

[2] Bortolotto, T. *et al.* Polymer-coated palladium nanoparticle catalysts for Suzuki coupling reactions. *J. Colloid Interf. Sci.* **439**,154-161 (2015).

[3] García-Suárez, E. J. *et al.* Efficient and recyclable carbon-supported Pd nanocatalysts for the Suzuki–Miyaura reaction in aqueous-based media: Microwave vs conventional heating. *Appl. Catal. A: Gen*. **468**, 59-67 (2013)

[4] Keesara, S., Mandapati, M. R. & Parvathaneni, S. Polystyrene supported salen type bis-thiopseudourea Pd(II)-complex catalyzed Suzuki coupling reaction in aqueous media. *Appl. Catal. A: Gen*.**2015**,*496*, 58-63.

[5] Edwards, G. A. *et al.* Melamine and melamine-formaldehyde polymers as ligands for palladium and application to Suzuki–Miyaura cross-coupling reactions in sustainable Solvents. *J. Org. Chem.* **79**, 2094-2104 (2014).

[6] Liu, C., Li, X., Gao, Z., Wang, X. & Jin, Z. In situ-generated nano-palladium-catalyzed ligand-free Suzuki–Miyaura reaction of potassium aryltrifluoroborates at room temperature. *Tetrahedron*, **71**, 3954-3959 (2015).

[7] De Castro, K. A. & Rhee, H. Resin-immobilized palladium nanoparticle catalysts for Suzuki–Miyaura cross-coupling reaction in aqueous media. *J. Incl. Phenom. Macrocycl. Chem. 82*, 13-24 (2015).

[8] Yuan, D. & Zhang, H. Nanosized palladium supported on diethylenetriamine modified superparamagnetic polymer composite microspheres: Synthesis, characterization and application as catalysts for the Suzuki reactions. *Appl. Catal. A: Gen.* **475**, 249-255 (2014).

[9] Wen, Q. *et al.* triptycene-based microporous organic polymer bearing tridentate ligands and its application in Suzuki–Miyaura cross-coupling reaction. *Macromol. Rapid Commun.* **36**, 413-418 (2015).

[10] Sun，X., Zheng, Y., Sun，L., Su，H. & Qi，C. Pd nanoparticles immobilized on orange-like magnetic polymer-supported Fe3O4/PPy nanocomposites: A Novel and highly active catalyst for Suzuki reaction in water. *Catal. Lett.* **145**, 1047-1053 (2015).

[11] Savva, I. *et al.* PVP-crosslinked electrospun membranes with embedded Pd and Cu2O nanoparticles as effective heterogeneous catalytic supports. *RSC Adv.* **4**, 44911-44921 (2014).

[12] Zhang, A. et al. Homogeneous Pd nanoparticles produced in direct reactions: green synthesis, formation mechanism and catalysis properties. *J. Mater. Chem. A,* **2**, 1369-1374 (2014).
[13] Uberman, P. M., Pérez, L. A., Lacconi, G. I. & Martín, S. E. PVP-stabilized palladium nanoparticles electrochemically obtained as effective catalysts in aqueous medium Suzuki–Miyaura reaction. *J. Mol. Catal. A-Chem.* **363-364***,* 245-253 (2012).
[14] Beygzadeh, M., Alizadeh, A., Khodaei, M. M. & Kordestani, D. Biguanide/Pd$(OAc)_2$ immobilized on magnetic nanoparticle as a recyclable catalyst for the heterogeneous Suzuki reaction in aqueous media. *Catal. Commun.* **32**, 86-91 (2013).
[15] Selivanova, A. V., Tyurin, V. S. & Beletskaya, I. P. Palladium Nanoparticles Supported on Poly(N-vinyl- imidazole-co-N-vinylcaprolactam) as an Effective Recyclable Catalyst for the Suzuki Reaction*. Chempluschem* **79**, 1278-1283 (2014).
[16] Mu, B., Li, J., Han, Z. & Wu, Y. Fast Suzuki–Miyaura cross-coupling reaction catalyzed by the Na2Pd2Cl6 complex with ethyl calix[4]aryl acetate at room temperature in aqueous medium under ligand-free and ambient atmosphere. *J. Organomet. Chem.* **700**, 117-124 (2012).
[17] Collins, G., Schmidt, M., O'Dwyer, C., McGlacken, G. & Holmes, J. D. Enhanced Catalytic Activity of High Index Faceted Palladium Nanoparticles in Suzuki-Miyaura Coupling due to Efficient Leaching Mechanism. *ACS Catal.* **4**, 3105-3111 (2014).
[18] Liu, L., Zhang, Y. & Wang, Y. Phosphine-free palladium acetate catalyzed Suzuki reaction in water. *J. Org. Chem.* **70**, 6122-6125 (2005).

Supplementary Table S2 Comparison of catalytic activities of supported Pd catalysts.

Catalyst	Pd loading (mol%)	Reaction time	yields
Pd/C	1	30min	55%
		1h	81%
		2h	95%
PdEnCat^{TM}40	1	1h	3%
		4h	78%
PCS2	0.01	2h	99%
	0.1	1h	99%
PCS1	0.001	2h	99%
	0.006	30min	99%
	0.01	5min	99%

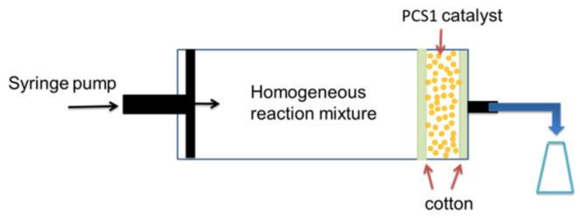

Supplementary Figure S3 Equipment for continuous flow Suzuki reaction.

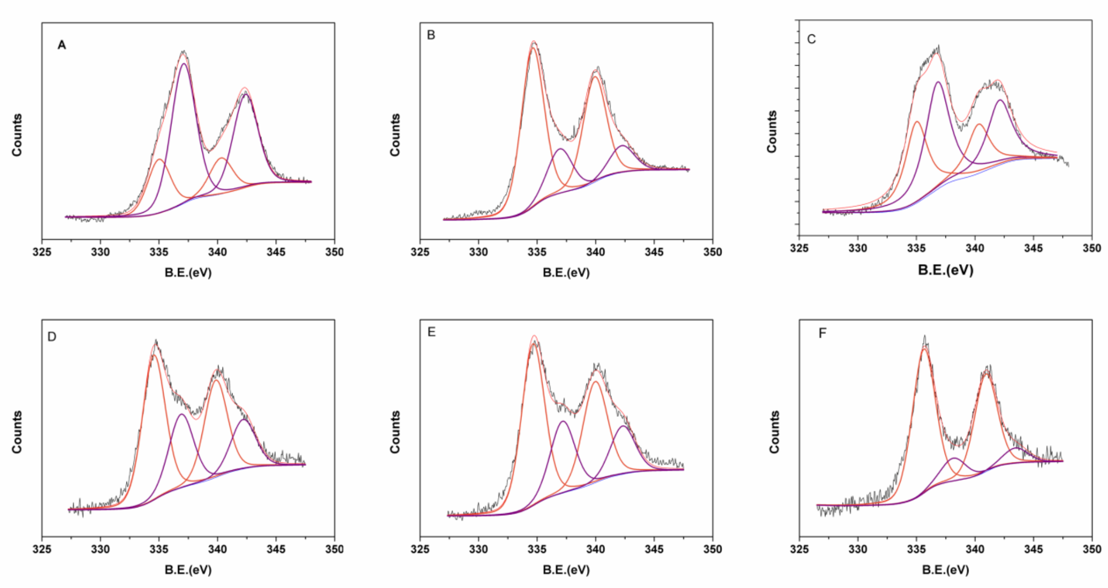

Supplementary Figure S4 XPS spectra of Pd 3d of as-prepared PCS1 (A), after 1st heterogeneous Suzuki coupling (B), kept in air after 1st heterogeneous Suzuki (C), after 1st homogeneous Suzuki coupling (D), after continuous flow Suzuki coupling (E), and after 1st Heck reaction (F).

Supplementary Table S5 BET results for macroporous PCS1 catalyst before and after the first Suzuki and Heck reactions.

Sample code	*PV*(ml/g)	S_{BET} (m^2/g)	D^a (Å)
PCS1 catalyst	1.91	32	2387
PCS1 after 1st Suzuki reaction	1.73	29	2386
PCS1 after 1stHeck reaction	0.89	27	1318

PV, pore volume; S_{BET}, internal surface; D, average pore diameter.

[a]Calculated from D=(4000PV/S_{BET}) (cylindrical model assumed)

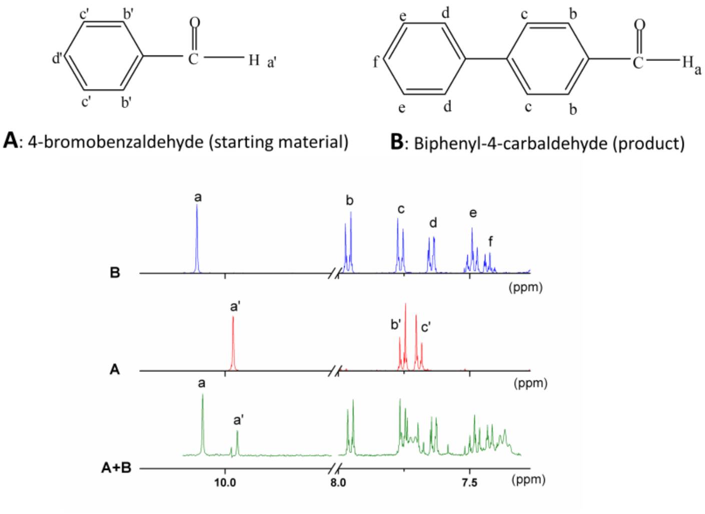

Supplementary Figure S6 Calculation of the conversion of 4-brombenzaldehyde) as the ratio of peak area $A_a/A_{(a+a')}$ ×100% in ^{1}HNMR spectrum (A+B).

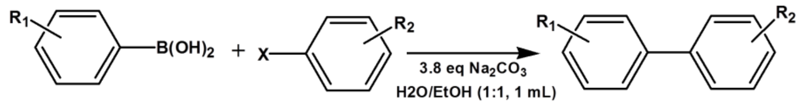

Aryl halide	Boronic acid	Time	Catalyst * PCS1 (0.01 mol%) Con. (%)	Catalyst * PCS2 (0.1 mol%) Con. (%)
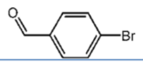	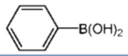	15 min	Qn.	Qn.
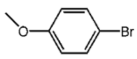	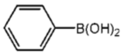	15 min	Qn.	Qn.
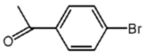	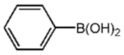	15 min	Qn.	Qn.
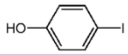	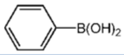	10 min	Qn.	Qn.
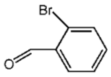	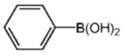	1h	Qn.	91.9.
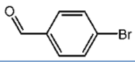	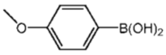	30 min	Qn.	Qn.
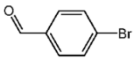	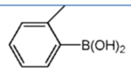	1h	96.6.	Qn.
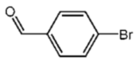	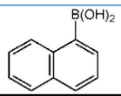	30 min	Qn.	Qn.

Supplementary Figure S7 Substrate scope of Suzuki cross-coupling reactions under concentrated heterogeneous reaction condition, (conversion was determined by ^{1}HNMR analysis based on aryl halides).

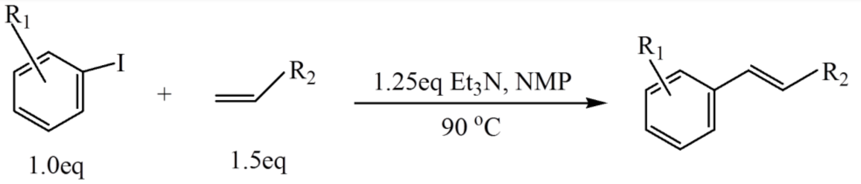

Entry	Catalyst[a]	R_1	R_2	Time (h)	Coversion(%) [b]
1	A	H	CO_2*n*Bu	2	>99
2	B	H	CO_2*n*Bu	2	>99
3	A	H	CO_2Et	2	>99
4	B	H	CO_2Et	2	>99
5	A	H	CO_2Me	2	>99
6	B	H	CO_2Me	2	>99
7	A	H	CO_2H	2	99
8	B	H	CO_2H	2	98
9	A	H	Ph	6	85
10	B	H	Ph	6	73
11	A	2-CH_3	CO_2Et	6	92
12	B	2-CH_3	CO_2Et	6	98
13	A	3-OCH_3	CO_2*n*Bu	2	>99
14	B	3-OCH_3	CO_2*n*Bu	4	>99
15	A	4- OCH_3	CO_2*n*Bu	2	>99
16	B	4- OCH_3	CO_2*n*Bu	4	88

A: PCS1 ; B: PCS2. [a]Pd loading: 0.6-0.65mol%.
[b]Conversion was determined by ^{1}HNMR analysis based on aryl iodides.

Supplementary Figure S8 Substrate scope of Heck cross-coupling reactions, (conversion was determined by ^{1}HNMR analysis based on aryl iodides).
